# From Self-Management to Shared-Management: A Relational Approach for Equitable Chronic Care

**DOI:** 10.1093/phe/phae007

**Published:** 2024-08-22

**Authors:** Francisca Stutzin Donoso

**Affiliations:** Department of Public Health and Primary Care, University of Cambridge, UK

## Abstract

Life with chronic disease and chronic care is hard and people who live in disadvantage may lack the freedom to prioritise their care because of increased competing demands. This paper proposes that shifting the goals of chronic care from self-management support to a shared-management approach can help improve wellbeing and health outcomes across social groups. This work draws on a qualitative exploration of the lived experience of chronic disease and an applied ethical analysis of the reproduction of disadvantages within chronic care. The qualitative results further specify respectful and collaborative patient-healthcare professional relationships; autonomy-supportive interventions; and continuity of care to face the complexity of chronicity in a phenomenological sense—a paradoxical experience of long duration that comprises the disease’s presence in the absence of its manifestation. The ethical analysis draws on performativity; autonomy and decision-making; and responsibility, which constitute the theoretical foundation for shared-management. This approach contributes to advance current normative thinking for health justice and outlines practical steps for its clinical implementation in the delivery of chronic care.

## Introduction

Much of the academic literature on chronic disease argues that failure to adjust to chronic disease is strongly associated with poor self-management (SM)—mainly understood as adherence to long-term treatment^1^, so there is a strong focus on self-management support (SMS) in chronic care (Bodenheimer *et al*., 2002; Ridder *et al*., 2008).A comprehensive review from 2003 conducted by experts from the World Health Organization (WHO) reported that adherence to long-term treatments was around 50 per cent in high-income countries and presumably comparable or lower in low-and-middle income countries, contributing to the high rates of premature death and poor individual and population health outcomes (Sabaté, 2003). Even though this report has not been updated, recent systematic reviews about this issue continue to report sub-optimal adherence worldwide, adding a possible social gradient in long-term treatment adherence (Ortego *et al*., 2011; Gast and Mathes, 2019; Ogungbe *et al*., 2021).

Health and care systems in the UK have been failing to offer adequate support for people living with chronic diseases (The King’s Fund, 2018) and relevant efforts to address this challenge are framed within the concept of co-production in the delivery of services (Coulter *et al*., 2013). Applied to the health sector, this concept focuses on the unique needs of people and regarding the ill person as an active expert in the consultation context, thus stressing the importance of patient-centeredness and involvement (Realpe and Wallace, 2010).

The generic care model for chronic disease in the NHS follows the ‘chronic care model’ (CCM), which is strongly focussed on productive interactions between practice teams and patients (Wagner *et al*. 2001; Realpe and Wallace, 2010). This model is based on Wagner’s (1998) research and following suggestions for reshaping ambulatory care for chronic illness in the US. Wagner (1998) highlights that the needs of people living with chronic diseases are diverse and that an acute care organisation and culture of care are unlikely to meet such needs. Furthermore, the CCM stresses the need for regular interactions with caregivers focussing on function and prevention of complications, and continuing follow-up initiated by clinical practice (Wagner, 1998). This suggests that chronic care and adherence to long-term treatment are dynamic and interconnected processes, instead of isolated interventions and a stable achievement respectively.

The CCM is a comprehensive approach that suggests adjustments to different aspects of the organisation and delivery of healthcare are necessary to meet the specific medical needs of people living with long-term conditions (Wagner *et al*., 2001). This model states that patients themselves become the principal caregivers and highlights the importance of SMS and education. Still, it also acknowledges that self-management education can only deliver problem-solving skills and improve health outcomes to some extent, specifying that sustaining these changes in the long term remains a challenge (Barlow *et al*., 2000; Bodenheimer *et al*., 2002). This might be associated with the multi-layered challenges faced by people living with chronic diseases, which often appear as trade-offs or competing demands among the disease, the self and life beyond illness, sometimes hindering healthful behaviours and SM tasks (Stutzin Donoso, 2020).

Health policies and programmes aiming to improve health outcomes for people living with chronic diseases in the UK are in theory aligned with the ideas around co-production. However, drawing on empirical research from the UK, Entwistle *et al* (2018) have argued that SMS, in practice, is narrowly understood as patient education for healthcare desired behaviour change. This is strongly focused on increasing treatment adherence to improve healthcare in terms of morbidity and mortality while reducing healthcare costs by reducing readmissions to hospital and multi-morbidity, for example, thus taking a rather instrumental view on patient input (Entwistle *et al*., 2018). Thus, there seems to be large agreement on ‘person-centeredness’ being the right approach but not on how this should look like in practice.

Such narrow or biomedically oriented approaches to SMS are ethically problematic for two reasons. The first is that they fail to fully acknowledge the distinction between helping people *manage chronic conditions*—in terms of symptom and disease control—and helping people *manage well with chronic conditions,* which aims at a more flexible goal comprising health, wellbeing and overall quality of life (Entwistle *et al*., 2018). The second reason is that narrow SMS approaches exacerbate the double inequity of chronic disease, namely that (i) even within UHC systems, there are significant health inequalities caused by differences in patients’ abilities to adhere to long-term treatments, and (ii) inequalities in long-term treatment adherence are correlated with, and aggravate, existing health inequalities (Stutzin Donoso, 2018). In part, this is because the better-off are more likely to find SM tasks straightforward and to have the resources to attend to or overcome neglected aspects of their care (Entwistle *et al*., 2018).

The ‘Expert Patient’ initiative, explicitly focuses SMS on ‘developing the confidence and motivation of patients to use their own skills and knowledge to take effective control over life with a chronic illness’ (Department of Health, 2002: 6) and aims at people achieving ‘good quality of life despite chronic disease’ (Department of Health, 2002: 9). However, building on moral discussions about illness, Edgar (2005) criticised this initiative for failing to acknowledge and support the skills that people living with chronic diseases need to make sense of their disease and the impact the disease has on people’s sense of self or identity. Charmaz’s (2002) work discusses the idea of habit and the breadth of people’s approaches as they attempt to re-construct their self-concept due to chronic illness. More specifically, issues around stigma and exclusion in chronic illness have been associated with the tension between Western values highlighting individual responsibility and self-sufficiency on the one hand and narrow accountability from the state on the other (Charmaz 2020). This resonates with further criticism of SMS paying little attention to the psychological and emotional issues faced by people living with chronic diseases and a lack of understanding of self-management as embedded within and facilitated by social contexts and support (Furler *et al*., 2008; Kendall *et al*., 2012).

Beyond its thin sense merely as long duration, chronicity of illness is also about how people position themselves both individually and socially in relation to the long duration of disease. Thus, considering chronicity in a phenomenological sense can help further specify the quality of the challenges raised by chronic disease at the level of experience and its potential impact on the daily demands of chronic care (Stutzin Donoso, 2022). Chronicity in a phenomenological sense stresses the ‘paradoxical experience of long duration of disease, which implies significant abstraction or awareness of its [the disease’s] presence in the absence of its manifestation, as likely to recur’ [ … ] demanding to ‘transfer the quality of continuous and irreversible to an experience that might be thought of as discontinuous in terms of how it is expressed in the body’ (Stutzin Donoso, 2020: 3). However, further research is needed to understand how the complexity of chronicity comes into play in the specific context of chronic care and the patient-healthcare professional relationship.

Building on an empirical ethics qualitative study completed in 2019 in the UK, this paper examines the patient-healthcare professional relationship in chronic care to identify the processes that can either help or hamper people living well with chronic disease in different contexts.

The methods section summarises the study design; the recruitment process; and the data generation and analysis. The results section is illustrated with excerpts from interviews and or clinical observation field notes. This section is split into three subsections, which highlight the normative value of the qualitative data that is presented in the discussion. The discussion focuses on the ethics of performativity; autonomy and decision-making; and responsibility. The normative claims presented in this section suggest a shift from ‘self-management’ to ‘shared-management’ in chronic care, underlining the need to acknowledge people’s interdependence and vulnerability. This approach contributes to advance current normative thinking around chronic disease health justice and outlines practical steps for its clinical implementation. This is argued to have the potential to significantly increase the chances of all individuals living with chronic disease to experience better outcomes and wellbeing.

## Methods

### Project Design

The overall project—*empirical ethics study on the lived experience of chronic disease in the UK*—was an interdisciplinary research project including an empirical element composed of a qualitative exploration of the lived experience of chronic disease and a philosophical element composed of applied ethical analysis of the reproduction of disadvantages within long-term treatment practices and the delivery of chronic care.

This paper focuses on and presents the analysis of the empirical and philosophical elements specifically around the patient-healthcare professional relationship in the context of life with chronic disease and chronic care.

For the purposes of this paper, the project included:

(a) a review of the literature examining chronic care and self-management support;
(b) individual interviews with people living with a broad range of chronic diseases exploring people’s experiences of chronic care and insights on what good—and bad—patient-healthcare professional relationships look like;
(c) participant observation of specialist healthcare appointments to provide context and support the interpretation of the interviews; and
(d) conceptual and ethical reasoning to examine the implications of life with chronic disease and the delivery of chronic care.

All research activities were led by a researcher with a background in psychology and experience in social sciences research and methods.

This project (IRAS ID 242301) had Health Research Authority (HRA) and Health and Care Research Wales (HCRW) approval. The Research Ethics Committee (REC) reference for this study is 18/LO/1548.

The research followed an iterative process where the philosophical analysis was interlaced throughout the study to guide, inform and respond to the empirical elements (a–c).

### Theoretical Framework for Data Generation and Analysis

This study subscribed to the idea that facts and values are unavoidably entangled (Putnam, 2002; Wilson, 2021). Within empirical ethics, integrative approaches acknowledge a hermeneutical component that implies looking at moral judgements as socially constructed in the sense that these largely depend on individuals’ understandings or interpretations and their experience within social practice and what is ethically required in specific contexts (Molewijk *et al*., 2004). More specifically, this study drew on a reading of phronetic social science (Flyvbjerg, 2001) as a way to conduct integrative bioethics research. This means looking at the issue of integration in empirical ethics from the hermeneutical lens of the ‘complex and always-morally-charged nature of every day sense-making’ (Carter, 2018: 2). This work aimed to transparently generate and draw on empirical data about the experience of participants to inform the normative discussion on how to improve equity for health outcomes for chronic diseases. Flyvbjerg’s (2001: 60––61) four value-rational questions^2^ for phronetic social sciences: (i) ‘Where are we going?’; (ii) ‘Is this desirable?’; (iii) ‘What should be done?’; and (iv) ‘Who gains and who loses; by which mechanisms of power?’ served as a methodological tool for ethical reasoning throughout the data generation and analysis.

#### Data generation

Adopting a qualitative ethnographic approach and conducting a normative case study was particularly well suited to produce the kind of detailed and contextual knowledge required for this study (Flyvbjerg, 2001; Thacher, 2006; Schram, 2012). A total of 27 semi-structured interviews and 27 participant observation sessions were conducted. The interviews offered a creative path for the expression of chronic illness narratives, which have a privileged status to approach the complexities and contradictions of real-life (Flyvbjerg, 2001). Participant observation sessions allowed for the generation of emerging data through the examination of several dimensions of a social situation simultaneously—physical, behavioural, verbal, nonverbal, and interactional in the context of broader social and physical environment (Gordon and Levin, 2015). The topic guide of the semi-structured interviews covered five main areas: (i) participants’ demographic information, (ii) participants’ sense of identity, their family background and youth; (iii) participants’ diagnosis and experience of chronic disease; (iv) participants’ thoughts and feelings about their future; and (v) participants’ experience of long-term treatment. This allowed for the generation of relevant empirical data according to the aim of learning about participants’ illness and healthcare experiences, as well as getting to know them beyond their illness (Sankar and Jones, 2015). This included learning about their activities and relationships, their past and their ideas about the future. Research on illness perceptions, which identifies relevant elements for coping in terms of self-management (i.e. symptoms, cause, consequences, and curability or controllability of disease, beliefs, attitudes, values and prejudice regarding treatment and the disease) helped structure and narrow down the topic guide (Weinman *et al*., 1996; Benyamini, 2012; Manriquez and Stuardo, 2015).

This paper focuses mainly, but not only, on the findings from areas (iii) and (v) and draws heavily on observation in clinics. Five people living with chronic disease who were members of the patient and public involvement (PPI) network at the University College London Hospitals Biomedical Research Centre helped improve the topic guide.

#### Data analysis

Participants’ chronic illness narratives were analysed via thematic narrative analysis looking at Landman’s (2012) four levels of analysis: linear (linguistic structure), relational (relationships), emotional (feeling, beliefs, values), and analytical (attributing meaning by drawing connections across narratives). The interviews were audio-recorded and transcribed verbatim. Field notes from the participant observation sessions were also transcribed and completed from memory within 24 hours of each observation. Data from the participant observation session facilitated gaining knowledge through first-hand observation of the social interactions of interest to this study, complementing and providing context for the interpretation and analysis of the interviews. A relational database was created using NVivo-12 software and all data was coded following an inductive process, which informed the thematic analysis (Landman 2012; Sankar and Jones, 2015). After completing the data generation process, an iterative feedback process helped refine the coding of the data and the emerging themes.

### Sampling, Recruitment and Consent

The inclusion criteria for this study specified English-speaking adults between the ages of 18 and 65 years, diagnosed with at least one chronic disease after the age of 18 years and prescribed at least one long-term treatment, namely at least one regular medication that required regular clinical review. In addition to these, further desirable criteria included the sample being heterogeneous in terms of socioeconomic and educational level of participants, and relatively balanced in terms of the number of male and female participants.

A total of 27 participants were recruited through four specialist care units based in two North Thames NHS Trusts hospitals (respiratory medicine; gastrointestinal disease; endocrine disease; and infectious disease) (Table 1). Participants were invited to take part in an audio-recorded interview of up to 1 hour and 30 minutes long and were asked to allow a researcher to join them in a healthcare appointment of their choice to observe and take notes. Participants signed a consent form before the research activities started.

**Table 1.** Characteristics of participants

	Total: 27
Age (years)	
Mean	46.6
Range	21–65
Gender	
Female	14 (51.9%)
Male	13 (48.1%)
Time living with a chronic disease diagnosis (years)	
Mean	15.4
Range	3–44
Diagnosis	
HIV	8 (29.6%)
Pulmonary fibrosis	5 (18.6%)
Crohn’s disease	4 (14.8%)
Type 1 diabetes	3 (11.1%)
Hormonal disorders associated with pituitary tumours	3 (11.1%)
Ulcerative colitis	2 (7.4%)
Asthma	1 (3.7%)
Lupus	1 (3.7%)
Region of origin	
United Kingdom	14 (51.8%)
Europe	5 (18.6%)
Africa	4 (14.8%)
Asia	2 (7.4%)
North America and the Caribbean	1 (3.7%)
Oceania	1 (3.7%)
Level of qualification	
Professional	19 (70.4%)
Non-professional	8 (29.6%)
Occupation	
Full-time or part-time jobs	21 (77.8%)
Unemployed	2 (7.4%)
Retired	2 (7.4%)
Full-time mothers	2 (7.4%)

Generating a nonprobability sample that fitted the purpose of the study did not need or justify a large number of cases, nor to determine the exact sample size in advance of the fieldwork (Neuman, 2014). Sample numbers in qualitative research can vary significantly and general recommendations suggest between 12 and 60 cases or participants, with 30 being the mean. Like many things in qualitative research, the final answer to how many participants or cases to include is that it mainly depends on the research purpose (Adler and Adler in Baker and Edwards, 2012). Following this rationale, this study aimed at recruiting a minimum of 12 participants and a maximum of 50 participants, considering that having more than one technique for generating data (interviews and participant observations) is often considered a significant variable to determine the number of participants since it enlarges and deepens the data emerging from the study, strengthening the outcomes of the study without the methodological requirement of increasing the sample (Adler and Adler in Baker and Edwards, 2012).

The final sample size was determined after conducting a first set of eight interviews and completing a preliminary analysis of the audio-recordings. Due to the richness of the data and the clear common tendencies across diagnostic groups—which informed the aim of the overall study, it was decided that a sample of between 25 and 30 participants would suffice to achieve the aim of the study.

Participant observation sessions involved a total of 19 clinicians (Table 2). Written consent was also obtained from clinicians to observe the healthcare appointments.

**Table 2.** Characteristics of clinicians

	Total 19
Gender	
Female	12 (63.2%)
Male	7 (36.8%)
Specialist unit	
Infectious disease	6 (31.6%)
Respiratory medicine	5 (26.2%)
Endocrine disease	4 (21.1%)
Gastrointestinal disease	4 (21.1%)
Training	
Consultant	9 (47.3%)
Specialist nurse	4 (21.1%)
Specialty Registrar	6 (31.6%)

## Results

As a normative case study, this work focused on contributing to the understanding of important public values, combining empirical observation with normative assessment (Thacher, 2006). The thematic analysis for the overall project led to five emergent themes. As mentioned in the methods section, the results discussed in this paper focus on the findings from the theme titled ‘Values and judgements around chronic care and the patient-healthcare professional relationship’. This analysis of the patient-healthcare professional dynamics in the context of chronic care and its centrality for chronic disease health outcomes is presented in three sections: ‘Good’ and ‘bad’ healthcare relationships: the power of assumptions and expectations’; ‘Working with what we have: chronic care as a shared activity’; and ‘Lifting the burden: continuity of care in chronic disease’. These sections highlight the normative value of the qualitative data, which is theoretically framed in the discussion.

### ‘Good’ and ‘Bad’ Healthcare Relationships: The Power of Assumptions and Expectations

Participants’ judgement and appreciation of chronic care varied but reflected the value of broader and truly person-centred aspirations. On a general level, most participants’ descriptions agreed on what made clinicians ‘good’ or ‘helpful’ in the specific context of chronic care. Rather than valuing qualities in themselves, qualities such as ‘politeness’, ‘empathy’ and ‘understanding’ were important insofar as they contributed to establishing and maintaining a ‘good’ relationship with a clinician. This meant that ‘good’ clinicians happened in the context of the relationship with a particular person living with chronic disease.

Similarly, participants valued clinicians’ expertise and knowledge alongside the ability to listen, namely putting the knowledge at the service of context. ‘Listening’ was understood in terms of validating participants in their experience and thoughts about their health, being open to negotiating and attending to their worries and questions. As exemplified in the next quotation, ‘good’ clinicians were expected not to conflate their experience and knowledge with the truth.

“[You] might see some [clinicians] that are quite arrogant and they are actually like ‘hey, I am the consultant here’ […] ‘I know’ whereas I haven’t found that here, I found them very much like they listen to me […] I trust them because they’ve got, you know? the experience, but […] they are not trying to force their views on me. They are quite happy for me to say how I feel, they are quite happy for me to suggest reasons behind my illness” (41-year-old woman living with Lupus).

During consultations, this was exemplified by clinicians dedicating considerable time inviting participants to share aspects of their lives beyond the illness. Besides this being important for clinicians getting to know participants and what mattered to them, this is central for chronic care because chronic diseases are dynamic and interwoven with life changes beyond illness, such as caring responsibilities or travel plans, which can directly affect disease outcomes and orient treatment. Participants asked for support on disease management, other illnesses, their overall health, life changes, and understanding emerging aspects of their illness. As seen in the next quotation, by having the opportunity to speak to a clinician about housing issues, the participant had the support she needed to show that mould worsened her asthma and eventually get her landlord to address the humidity issues in her house.

“He is [a] good listener even if I speak to him about my house problem he writes for me a certificate to the environment department, for example, he is a good support my GP” (56-year-old woman living with asthma).

More generally, some participants described that when healthcare teams had a ‘good attitude’ in terms of being ‘friendly’ and ‘caring’ this motivated them to adopt a similar attitude. As exemplified in the next quotation, this positive dynamic was argued to contribute to building a working partnership for treatment.

“They are great, I love the nurses, super helpful, very risk-based approach and just really nice people, like they are really kind, even though they are super busy they are never rude, they are never crossed, they are never short with you.

### Interviewer: Do you think this has an effect on your care?

Yeah, because I am able to ask them things and I am not worried about asking them, yeah, so they are super professional.” (29-year-old woman living with Crohn’s disease).

In contrast, ‘bad’ or ‘unhelpful’ clinicians were mainly associated with having experiences of testimonial and hermeneutical difficulties. Such difficulties included clinicians’ lacking empathy and emotional investment, being uninterested and failing to take people living with chronic disease seriously by showing unwillingness to consider their worries or questions and making unilateral decisions about their care. In the next quotation, the participant describes a healthcare interaction that, although well-intentioned, disregards the participant’s context and suggests a path of action that significantly affected his wellbeing. The participant had been signed off work and socially isolated for over 7 years due to his illness.

“I said ‘ahh I work part-time’, why do you do that? [asked the doctor] because I like to go to the gym and try and stay healthy because of my, you know? weight loss, I said […] plus I am trying to make up on life, plus I get quite tired…‘you should be able to work full time’ [replied the doctor], ‘I think you should work more hours’ [said the doctor] I actually went and worked more hours because I felt shit, I was like ‘oh crap, I am just being lazy just like I was before’, ‘maybe I am not pushing myself’. Actually, that was a real struggle at that time, I wasn’t ready for it” (31-year-old man living with pituitary disease).

Overall, participant observation sessions suggested that many participants felt anxious or stressed before and during appointments. Some signs of this included participants coming in very early, bringing a written list of questions for the clinicians or carrying a folder with medical documents possibly to avoid forgetting important information, feeling more confident and or being taken seriously.

Some participants shared negative views about clinicians who did not consider their personal history, going through standard motions that did not apply to them, and disbelieved them or questioned their knowledge of their body. Many participant observation sessions offered good examples of some of these elements, showing little receptiveness from clinicians who mostly focused on completing administrative tasks on the computer. In these appointments, there was little eye contact and communication was difficult, clinicians asked few open questions and tended to interrupt participants. This feeling of clinicians being disconnected from participants’ care is exemplified in the next quotation.

“I actually find that [in] most appointments [clinicians] just don’t care, I walk in and they just ask real generic crappy questions ‘how do you feel?’ Fine, ‘cool’…yeah ‘see you in 6 months?’ Yeah, cool, no problem…I am out of there in 10 minutes” (31-year-old man living with pituitary disease).

In sum, this section described ‘how’ (rather than ‘what’) relational qualities can either support or hamper ‘good’ relationships between people living with chronic disease and healthcare professionals engaging in chronic care. Rather than specific qualities, participants’ experience suggests that what matters is the genuine intention to acknowledge the other as an individual and to establish a relationship. This specifically highlights the importance of validating and respecting people’s views, context and circumstances to share relevant information and devise helpful treatment options.

### Working With What We Have: Chronic Care as a Shared Activity

Many participants described that making informed choices about their disease and long-term treatment was difficult and experienced frustration about the lack of answers. Some participants described learning to deal with their condition through trial and error rather than following clinicians’ ‘textbook’ recommendations, as individual variability was significant even within diagnostic groups. Participants thought that ‘good’ patient-healthcare professional relationships reflected the value of clinicians caring for them in particular, suggesting that being treated in a sensitive and responsive way is important in chronic care.

Some participants reflected on the fragile epistemic status of medical truths about chronic disease in particular because of the uncertainty about recovery after acute episodes of ill health and the overall prognosis of their disease.

“When I go see the endocrinologist it’s always tiny bits of adjustments to improve the tiredness, but you know, so I am always reading and understanding and asking lots of questions and try, you know trying little things to improve that it’s kind of a little bit hopeless because I don’t think there’s much answer” (54-year-old man living with pituitary disease).

Thus, participants’ sources for deliberation in decision-making also included people outside medicine who helped them think about their circumstances and preferences rather than about the medical information. In other words, assess what would work best for them in particular.

Closely linked to the unavoidable uncertainties underlying chronic disease and chronic care, some participants described that the disease and or treatment were not properly explained to them, resulting in confusion especially regarding the consequences and prognosis of the disease. Examples of this included receiving misleading information about the expected outcomes of surgery (often curative), and clinicians avoiding questions about the cause of disease instead of acknowledging that, in many cases, there is not a straightforward answer. In the next quotation, the participant describes how she felt about having an ‘umbrella diagnosis’ with no explanation of the long-term consequences, even though she had been receiving regular specialist care for over five years.

“I don’t feel good about it because if there is no answer, how could you get a cure? […] I know she said to me I might, I might have to be on this medication for a long period of time…yeah […] I need to ask her [consultant] to find out exactly what it is because the last time I spoke to her [consultant] she said […] lung disease, but when I spoke to the nurse she said to me something different [pulmonary fibrosis] and I was like, what? And she said to me she is gonna send information in the post, I haven’t gotten any yet” (46-year-old woman living with pulmonary fibrosis).

In this context, participants valued feeling listened to, safe, understood and reassured, trusting that their healthcare team was focused on finding ways of making things better when problems arose, instead of assigning blame. A few participants highlighted how harmful it can be for the relationship and its potential for collaboration to be judged and blamed by clinicians, or as exemplified in the next quotation, even reprimanded after a bad test result.

“It’s not helpful you know? it’s hard to get [diabetes] and have to live with it and then get a bad result and then someone trying to give out to you like you are a child, it’s like, you know? I remember feeling ‘oh I really don’t’, I can’t even put a face on that doctor, but I do remember I was cursing him when I got out” (30-year-old woman living with type 1).

Chronic disease manifestations can often be beyond the individual’s control and genuinely hard to balance, as largely affected by contextual variables. Thus, when the clinician in the last quotation handed responsibility for poor outcomes over to the participant, this was experienced as unfair or too harsh. Despite how controlled or out of control the disease was, many participants described themselves as relatively ‘good patients’, meaning that they did everything they could to take care of themselves even if this did not always mean following clinicians’ recommendations to the letter.

Highlighting the role of collaboration in chronic care, many participants described that it was important that the specialist team was somehow available in case they needed to contact them. This need is directly linked to how difficult it can be to anticipate and manage a chronic disease flare-up or complication. Having an available and responsive team made participants feel validated and more in control when the disease spun out of control. Participants valued the clinical team being reachable (via email or phone), responsive and ready to empathise with their situation. As seen in the following quotation, this allowed them to make timely decisions together.

“So, when it flares-up I will then, you know? I may…I often initiate a course of steroids, I would phone them [hospital team] up and I’ll say ‘I have done this’ and they will say ‘yes, that’s fine’ and together we’ll work out what to do for the week” (49-year-old woman living with rheumatic disease).

It is rather straightforward how in the context of chronic care clinicians knowing the person living with chronic disease and her broader circumstances can help provide better healthcare recommendations, but participants’ experience also highlighted how people living with chronic disease knowing the clinician was particularly important in chronic care. A few participants stressed how getting to know clinicians helped them anticipate how they would care for them outside the consultation room, helping participants gauge how much of the burden of organising their continuous care might fall back on them. Working with a proactive clinician means that crucial tasks are completed without the person living with chronic disease having to follow-up on them. The next quotation is a good example of the level of proactivity and efficacy required from people living with chronic disease when crucial tasks in their care seem to fall through the cracks:

“I have worked out a system for it now…but in the beginning that was difficult because the GP would give out to me for not having the blood test results and I would say, ‘but I don’t know how to get them’ and she said ‘just email the hospital or tell them’ and then I was like…‘OK, fine’ […], ‘I have to take responsibility for this, because there’s a gap in the handover between the hospital and the GP” (29-year-old woman living with Crohn’s disease).

In sum, this section described how the limitations of medical knowledge about chronic disease trajectories stress, even more, the importance of focusing on individual rather than ‘diagnostic categories’ and understanding that chronic care will often involve knowledge and resources beyond biomedicine. Moreover, and because of the intrinsically unpredictable nature of chronic disease, a ‘good’ patient-healthcare professional relationship in this context values individuals’ efforts beyond healthcare outcomes, and is responsive and forward-looking, specifically in terms of the responsibility to solve problems as they arise.

### Lifting the Burden: Continuity of Care in Chronic Disease

Participants stressed that a ‘good patient-healthcare professional relationship’ allowed for discussion and agreement over treatment approaches and goals. Feeling that communication was fluent and easy was argued to be very important to achieve this, and this, in turn, was facilitated by continuity in the relationship with a known clinician:

“I think they [known clinicians] give you better advice because they know what you are prepared to do and what you are not prepared to do, and you know? they can kind of develop that relationship with you and work with you […] it’s quite hard when you only got whatever they are 10-15 minutes appointments to really address someone’s long-term overarching condition if you don’t have any understanding of where they are at” (36-year-old woman living with type 1 diabetes).

Although continuity of care seemed less relevant and the relationship merely instrumental when treatments were particularly straightforward, participants were still critical of the lack of consistency that resulted from isolated appointments. This was closely intertwined with the idea that different clinicians could often have different approaches to treat the same condition. This is highlighted in the next quotation.

“Different consultants have different ideas about treatment so you can see one and they might put you on something and then you see another, and they might wait, and I think that’s important, to have the same view if you are happy with that consultant” (41-year-old woman living with Lupus).

During the participant observation sessions some participants from all disease groups not only had appointments with clinicians they did not know, but they also sometimes saw someone different from whom they were expecting to see. Often, this made participants feel disappointed with the care they received, as appointments reflected mainly instrumental exchanges. These encounters lacked a long-term plan and shared goal for treatment, mainly focusing secondary care for chronic diseases on flare-up management and offering little support regarding overall wellbeing and quality of life.

Specifically in secondary care, participants described continuity of care as having a consultant who is somehow in charge of their care and getting to see this consultant regularly but not necessarily every time. Seeing a different clinician for regular check-ups, especially in the context of teaching hospitals with rotating professionals, was not a problem for some participants if they could see their known consultant upon request, as lack of continuity was still described to affect collaboration and limit the scope of appointments.

The relationship with the consultant was so important for some participants that they chose to travel from other cities to see them or worried about what will happen to them when their known consultant retires. The next quotation reflects the special value of continuity of care in chronic disease treatment because of the variable experience of control described in the previous section.

“I really trusted her [consultant] and I think it was reassuring for me to always see her because she knew me and I didn’t have to repeat the whole story in each consultation whereas now I think, as my illness has become more controlled, don’t really mind who I see but if one day […] it became more difficult to control I bet I’ll want to see her again” (49-year-old woman living with rheumatic disease).

Although continuity of care was similarly important in primary care, participants described that it was difficult to see the same GP both because of the limited availability of appointments and continuity not being prioritised in how appointments were allocated. Thus, unless participants ‘got lucky’, seeing the same GP implied waiting weeks or months, which is often incompatible with primary care needs. Continuity of care in primary care was argued to facilitate communication and provide the necessary context to address issues properly.

Some participants described little gain from seeing GPs and described these appointments as frustrating encounters. GPs were described as ‘clueless’ regarding participants’ treatment and considerations around their main chronic disease diagnosis. This revealed a certain degree of tension between different levels of care, putting participants in a difficult position as mediators and, sometimes, lone advocates of their care.

The tension between levels of care also came up in many participant observation sessions, as the appointment became a space for participants to complain about difficulties coordinating their GP care, sometimes asking the consultant to take action. Some consultants also expressed their frustration about GP management of some elements of care that affect long-term treatment. Participants tended to centralise their care at the specialist level, often asking for the consultant’s opinion on something that was ‘GP territory’. This underlines how important it was for participants to share the responsibility for their care by having someone overseeing and, at least to some extent, integrating the care. The next quotation reflects the value of integration both between services and within services, so ideally different clinicians (i.e. nurses and consultants) within the care team can all understand the context of care and offer consistent and helpful clinical advice.

“I was having a really hard time with bleeding and stuff and one of them [nurses] because she knew all the situation properly and stuff, like, she gave me a certain enema that I could take, that really helped me and rather than when I went to the doctor, all he could say was ‘yeah, you need surgery’. So, it’s like when someone listens and takes the time to think sometimes it’s easier to just find solutions” (21-year-old man living with Crohn’s disease).

In sum, this section described how clinicians who cared about people living with chronic diseases personally and knew what mattered to them had enough contextual information to tailor their approaches in such a way that treatment considered the complexity of people’s personal circumstances, achieving better outcomes.

Although continuity of care cannot ensure better outcomes, it is difficult to imagine good outcomes without some form of continuity of care.

## Discussion

This section discusses the conceptual and ethical implications of the empirical results presented in the previous section. More specifically, the theoretical analysis presented in this section is the conceptual framework for the normative outcomes of this work.

As an integrative empirical ethics study, the information presented in the results and the discussion is artificially split into sections and subsections to facilitate the analysis. Even though the headings are not the same (responding to different levels of analysis), the subsections in what follows build on and map onto the subsections in the results. Thus, mainly drawing on:

1) Participants’ descriptions of ‘the power of assumptions and expectations’ in relation to ‘good’ and ‘bad’ patient-healthcare professional relationships, the first subsection of the discussion will reflect on ‘the ethics of performativity’ to reflect on the mechanism by which patient-healthcare professional relationships may counteract or contribute to reproduce pre-existing inequalities in chronic disease.
2) Participants’ descriptions of ‘chronic care as a shared activity’ with healthcare professionals and beyond, the second subsection of the discussion will reflect on what this means for ‘autonomy and decision-making’ in the context of chronic care. This will suggest adopting a view of autonomy as socially constituted and shared decision-making practices that will be autonomy-supportive if they focus on individuals’ needs and context.
3) Participants’ experience of ‘continuity of care in chronic disease’ and its potential to facilitate collaboration for a ‘good’ patient-healthcare professional relationship that can work towards shared treatment goals, be autonomy-supportive and counteract health outcomes inequalities, the third subsection of the discussion will reflect on what this means for ‘responsibility’ in chronic care. This will highlight the value of adopting a forward-looking shared responsibility approach that goes beyond desertism and luck egalitarian views to work towards chronic disease justice.

### The Ethics of Performativity

What participants valued and did not value in their relationship with clinicians is closely linked to the egalitarian principle of ‘being treated as an equal’, this is, being treated with equal concern and respect, where respect means for people to be treated as ‘capable of forming and acting on intelligent conceptions of how their lives should be lived’ (Dworkin, 2013: 326). Furthermore, the first subsection of the results highlighted that how people living with chronic disease were treated evoked different responses, suggesting a performative effect in the patient-healthcare professional relationship that worked both for good and bad. When participants felt they were being treated with equal concern and respect, they felt compelled to treat the system and healthcare professionals in a similar way, establishing positive relationships where mutual trust allowed for fruitful collaboration. In contrast, when participants did not feel they were being treated in this way, significant resistance arose against the system and individual healthcare professionals as well as discontent with the treatments offered, disengagement with care and poorer outcomes in terms of wellbeing.

Describing this process in terms of a performative effect draws on Biggs’ (2011) analysis of performativity and self-fulfilling prophecy (SFP), which is when a belief comes to be true because people act as if it is (Biggs, 2011). In the context of healthcare systems, Wilson (2021) refers to complex systems’ performativity to describe how people’s interpretations and others’ expectations within a system can make it easier or harder for someone to achieve a desired goal, thus changing the behaviour of the system.

Applied to chronic care, this would suggest that healthcare professionals’ explicit or implicit assumptions about people living with chronic disease (as worthy of equal concern and respect or not), will evoke behaviours that can make such assumptions come to be true, changing the behaviour of the system. As discussed in the results, the ways in which the system behaves following this process can either counteract or reproduce pre-existing inequalities. Reflecting on Biggs (2011) plausible explanations for SFPs in more detail will help unpack this statement. Based on careful analysis of social studies, the first explanation draws on the example of a teacher–student relationship. This explanation argues that a teacher’s belief that a student is mediocre can lead to the student’s mediocre results because this may affect the student’s motivation or because the student unconsciously fulfils the teacher’s expectation. The second explanation draws on the example of a social group, assumed to be violent, responding with violence to the police’s violent repression. The third explanation draws on the example of people who believe they are mistrusted being less likely to behave in a trustworthy way, which suggests that people can intentionally live up or down to other’s expectations (Biggs, 2011).

Although this is not discussed by Biggs (2011) it seems rather straightforward that, like in the patient-healthcare professional relationship, power relations play a role in how each of these outcomes come to be true, and thus in the dynamic process of SFPs. Acknowledging power dynamics can help explain even why someone ‘unconsciously’ may fulfil expectations in social contexts. A student is more likely to accept a teacher’s belief about her academic ability over her own because of the expected knowledge gap in that relationship—similar in this sense to the patient-healthcare professional relationship; accepting or rejecting the police’s assumptions does not make a difference because of the power invested in it by the state to judge the situation; and although the last example is unspecific, it might be argued that whose expectations are fulfilled and whose are not might depend, to some extent, on epistemic trust, namely whether the person or group holding the expectations is trusted as someone who can introduce new knowledge about the self (Allison and Fonagy, 2016).

Taking this further, and also drawing on Young’s (2011) idea that people act within institutions where they know others have certain expectations of how things are done and individuals react with sanction if the implicit or formal rules are violated, it might be argued that remaining unaware of social hierarchies and encountering people living with chronic disease with narrow and static expectations because of their social context, level of education, background or past behaviour, for example, is likely to shape their health behaviours. Perhaps clinicians’ most likely starting point is to—more or less—consciously agree with the idea that everyone deserves equal concern and respect. Still, it can be hard to remain self-critical and resist the explicit or implicit labels (beliefs) of ‘good’—compliant—and ‘bad’—non-compliant—‘patients’ and therefore have these assumptions shaping healthcare outcomes for chronic care. This information is important for clinical judgement and care, just not in such a linear way. Assuming that someone who follows long-term treatment recommendations will continue to do so and someone who does not will continue not to, fails to understand the dynamism of the illness and treatment processes (Stutzin Donoso, 2020) and may play an important role in both outcomes coming to be true.

Because the worst off have to risk the prioritisation of chronic care in a way that the better-off do not have to (Stutzin Donoso, 2018), the former are more likely to be labelled as ‘bad patients’ and the latter as ‘good patients’, so introducing the idea of performativity in the patient-healthcare professional relationship helps further specify the mechanisms underlying the reproduction and amplification of existing health outcomes inequalities among people living with chronic diseases. As discussed in the results, because of their social role and epistemic power, clinicians’ practices of testimonial justice and participatory prejudice (Kidd and Carel, 2017), may respectively help compensate for prejudice and prevent further disadvantage or augment experiences of prejudice, risking further disadvantage by undermining the precarity of chronic disease management.

### Autonomy and Decision-Making

As presented in the second subsection of the results, participants described how chronic care requires a kind of collaboration with healthcare professionals and the wider social context that is sensitive to the contingency of chronic disease. Understanding chronic care as something that clinicians and patients do together—in the way co-production and person-centred care encourage—implies that each of their actions is part of a whole that neither of them can do on their own (Walker, 2019). Still, this might be seen as an ethically problematic territory, as collaborative relationships may challenge traditional or narrow ideas of autonomy and paternalism. Such ideas do not consider the sustained relationship that emerges in the context of chronic disease, even if only through patients’ records. Clinicians often worry about finding a balance between being accessible or responsive, while making sure that patients do not become too reliant on them or the service (Owens *et al*., 2017). Still, chronic disease implies being physically vulnerable and dependent (Stutzin Donoso, 2020) and, as seen throughout this study, it also means heavily relying on healthcare services, clinicians and treatment to stay alive and or manage well with chronic disease.

From a narrow healthcare ethics perspective, clinicians have the main responsibility of benefitting the patient while respecting their autonomy. This has been thought to imply that any actions beyond providing patients with the knowledge and resources necessary to manage their illness might be regarded as unacceptable on account of paternalism, and further harm would be the patient’s responsibility (Walker, 2019). However, consistent with the results of this study, the main benefits of treatment in chronic disease have been described as ‘(i) alleviating or preventing subjectively unpleasant experiences, and (ii) counteracting the ways in which the patient’s illness negatively affects, or would if untreated negatively affect, her ability to live her life in her own terms (either now or in the future)’ (Walker, 2019: 134).

In line with managing well with chronic disease and treating patients as equals, the second aim in the last quotation combines beneficence and autonomy as the capacity for self-governance, this is, being able to make decisions about one’s life free from the interference of others (Owens and Cribb 2013; Walker, 2019). However, long-term treatment is complex and it can often affect people’s lives in similar or worse ways than the disease itself. One of the complexities discussed in this study is that despite significant advances in medicine, the lack of medical knowledge regarding best courses of action continues to be an important consideration in chronic care (Toombs, 1987). As presented in the results (second subsection), many participants in this study described aspects of uncertainty regarding their health and treatment, sharing an overall sense that medical knowledge for their conditions did not seem to be precise.

From the perspective of clinicians, this translates into significant moral stress, as they experience the pressure of dealing with the question of what may be the best path of action for a patient in a context where healthcare is mainly seen as curative and hopeful, thus making the delivery of chronic care especially hard (Berlinger, 2016). As exemplified in the results of this study, in the face of morally troubling decisions or difficult conversations, clinicians tend to display workaround mechanisms that implicitly or explicitly transfer the responsibility to patients (Berlinger, 2016). In part, this may be harder than necessary because autonomy in healthcare tends to be reduced to decision-making. In this context, and from the perspective of clinicians, practices of ‘shared decision-making’ are those that include patients in medical decisions that affect them, focusing on offering information to support and respect patients’ autonomy and avoiding recommendations that can interfere with their deliberations (Owens and Cribb, 2013). However, this downplays the potential positive effect that collective deliberations can have over patient autonomy (Owens and Cribb, 2013).

In trying to be autonomy-supportive, clinicians might give insufficient or unclear information, which would have the opposite effect. Focusing on respecting patients’ choices in a narrow sense often means ‘stand-back and don’t interfere’, which makes patients feel like their doctors refuse to use their expertise to guide them (Entwistle *et al.*, 2012). Broader understandings of ‘shared decision-making’ focus on clinicians and patients engaging in meaningful processes, highlighting the value of clinicians drawing on their expertise to flexibly offer guidance and recommendations according to individual patients and situations (Entwistle *et al*., 2012). The positive effect of such practices was reflected in the results section when discussing how working together with a clinical team gave patients an increased sense of control. Such shared decision-making practices build on a different understanding of autonomy, namely ‘autonomy’ as a socially constituted capacity, which falls under the umbrella term ‘relational autonomy’ (Mackenzie, 2008). Drawing on this, Owens and Cribb (2013) have argued that decision-making processes in healthcare are often influenced by people’s context and specific circumstances. Hence, as seen in this study, clinicians’ recommendations are likely to be autonomy-supportive if clinicians listen to patients; take their context and circumstances into account; allow for questions and corrections about their understanding of the patient and ensure that patients can choose against their recommendations without seeing their care being affected (Entwistle *et al*., 2010). Furthermore, building on the performativity argument, being autonomy-supportive in this way works as an operationalisation of ‘equal treatment’ in the sustained relationship emerging within chronic care, having the potential to stop or slow down and counteract pre-existing health outcomes inequalities.

### Responsibility

The discussion thus far has been circling around the idea that moving beyond individual roles and responsibilities allows focusing on that, ultimately, the success or failure of chronic care will depend on clinicians and patients collaborating, this is, each doing their part in the shared project or activity of chronic care (Walker, 2019). In addition to this, participants’ views presented the third subsection of the results highlighted the temporal dimension of this shared activity by introducing a broad or flexible understanding of continuity of care. Participants described chronic care as something that clinicians and patients do together beyond healthcare appointments, as how they acted separately but in a coordinated way between consultations mattered as much or even more than seeing a known clinician. This has been conceptualised by Walker’s (2019) work on the ethics of chronic illness and complements the two core features across different conceptualisations of continuity of care—namely a ‘continuous caring relationship’ with a clinician and a ‘seamless service’ as coordinated care both between and within services (Gulliford *et al*., 2006)—by also considering the patient’s role in this coordinated effort.

Thinking about chronic care in these terms suggests that responsibility for its outcomes is shared. This means that although patients are responsible for their circumstances to some extent, what they are owed should not depend on the decisions they make and if they do not follow treatment recommendations, for example, they should not have less priority or be punished in any way (Walker, 2019). This argument is part of a larger and still active debate on the applications of luck egalitarian views of justice to support rationing and incentive policies for health justice (Voigt, 2013). Such ideas seem to suggest that the ‘brute luck/option luck’ framework may be used to establish desert as an approach to fairness, but this is in fact at odds with desertist views of distributive justice, where what people are owed depends on their contribution regardless of how it came about (Brouwer and Mulligan, 2019). In this sense, desertism may be more flexible than luck egalitarianism for reconciling issues already discussed in this paper around the cumulative effect of disadvantage affecting those who live with chronic disease. Yet, the idea of shared responsibility for chronic care seems to push even further and suggest leaving the rationale of desert behind to think about chronic disease justice.

Clinicians and patients who operate on the basis of accountability arguably hold a luck egalitarian view of justice, no matter how difficult this might be in the context of their required collaboration. Luck egalitarianism sees bad outcomes resulting from option luck individual choices—e.g. risks people voluntarily accept such as those resulting from not following or partially following treatment recommendations—as the individual’s own responsibility (Voigt, 2013). However, Voigt (2007) has argued that despite taking context into account—the influences of unequal bad brute luck on individual’s choices—luck egalitarianism is too harsh to deny compensation because this unjustifiably limits people’s freedom to take risks. Similarly, but arguably even more morally problematic, it seems too harsh to blame and potentially punish people living with chronic disease for poor health outcomes considering that they have to make health decisions within significant uncertainty while also being likely to be in the position to have to take risks—lacking the freedom not to take risks—to negotiate competing demands that exceed the management of the disease.

As seen in the results, participants’ intuitions around responsibility for long-term treatment focused on working together to move forward when flare-ups or complications arose, mirroring a forward-looking conception of responsibility that does not assume blame, fault or liability as the main way of assigning responsibility (Young, 2011). This different kind of responsibility does not undermine the fact that individual action and choice contribute to someone’s circumstances but derives from the fundamental idea that people belong ‘together with others in a system of interdependent processes of cooperation and competitions through which we seek benefits and aim to realise projects’ (Young, 2011: 105). This means that shared responsibility is something people personally bear, but they do not bear alone. Building on the results of this study and the intertwined philosophical analysis, embracing an understanding of responsibility as always personal and shared is central to a framework of care that can promote justice throughout processes and outcomes in chronic disease. This subsection has argued that shared responsibility can help operationalise ‘equal treatment’ and contribute to its implementation in clinical settings.

## Conclusions

The results of this study suggest that the current focus on ‘self-management’ of chronic disease (i) limits patients’ outcomes and wellbeing and (ii) contributes to perpetuate chronic disease health outcomes inequalities. Based on the analysis of patients’ experience of chronic disease and chronic care, the philosophical discussion in this paper focused on the ethics of performativity; autonomy and decision-making; and responsibility. This applied ethics process led to the development of a ‘shared-management’ approach to chronic disease that specifies how: (i) respectful and collaborative patient-healthcare professional relationships; (ii) autonomy-supportive interventions; and (iii) continuity of care should look like to improve health outcomes for chronic disease across social groups. This approach contributes to advance current normative thinking around chronic disease health justice and outlines practical steps for the clinical implementation of the egalitarian principle of ‘being treated as an equal’.

The ‘shared-management’ elements described here are preliminary and would benefit from testing and further specifying. Still, the approach described offers solid grounds and clear guidance to embrace the ongoing challenges of living with chronic disease and long-term treatment, recognising the process, its frailty and the different needs for continued support. This has the potential to significantly increase the chances of all individuals living with chronic disease to experience better outcomes and wellbeing.

## Footnotes

1 This concept is being used descriptively to refer to individuals taking treatments available to them that, from a biomedical perspective, could help improve their health outcomes. This does not suggest, however, any normative claims about whether individuals *should* or *shouldn’t* take such treatments.

2 These questions are a method and not an aim in itself, so the study did not focus on providing full answers for these specific questions.
